# Irisin Exerts Neuroprotective Effects on Cultured Neurons by Regulating Astrocytes

**DOI:** 10.1155/2018/9070341

**Published:** 2018-09-26

**Authors:** Kexin Wang, Hongyan Li, Hongxing Wang, Jun-hui Wang, Feng Song, Yu Sun

**Affiliations:** ^1^Department of General Surgery, Qilu Hospital of Shandong University, Jinan, Shandong, China; ^2^Department of Obstetrics and Gynecology, The Eighth People's Hospital of Qingdao, Qingdao, Shandong, China; ^3^Department of Neurology, Xuanwu Hospital, Capital Medical University, Beijing, China; ^4^Department of Physiology, University of Toronto, Toronto, Ontario, Canada; ^5^Department of Orthopedics, Qingdao University Affiliated Qingdao Municipal Hospital, Qingdao, Shandong, China; ^6^Department of Endocrinology, Qilu Hospital of Shandong University, Jinan, Shandong, China

## Abstract

Neurons suffer detrimental effects from *β*-amyloid toxicity in Alzheimer's disease. The exercise hormone, irisin, is found to induce a neuroprotective gene program and facilitates the beneficial effects on cognitive function. But no effort is made to test its direct protective effects on neurons against the A*β*-induced cell toxicity so far. In the present study, we investigated whether irisin could protect neurons against A*β*- (25–35) induced cell damage and explored the possible underlying mechanisms. Primary cell cultures of astrocytes and neurons were established. Conditioned medium from astrocyte was collected for the treatment and biochemistry assay study. To explore the protein expression changes, Western blot and ELISA assays were used in these *in vitro* cell culture models. Exposure of hippocampal neurons to 10 *μ*M A*β* (25–35) caused significant reduction on cell viability, and the toxic effect was not significantly reduced by the coadministration of irisin. However, pretreated astrocyte-conditioned medium with irisin for 12 hours notably protected the neurons from the toxicity of A*β*. Also, we found that irisin could attenuate the release of IL-6 and IL-1*β* from cultured astrocytes and decrease the expression level of COX-2 and phosphorylation of AKT. Last, we found that irisin could reduce NF*κ*B activation in astrocyte exposed to A*β* by preventing the phosphorylation and the loss of I*κ*B*α*. Our finding may provide novel evidence for the future application of irisin in the treatment of Alzheimer's disease and the memory dysfunction in diabetes mellitus.

## 1. Introduction

Alzheimer's disease (AD) is a progressive neurodegenerative disorder, which is characterized by extracellular amyloid plaques and cytoplasmic tau tangles [1]. Although progress has been made of understanding this devastating disease during the recent decade, AD remains incurable and is still a major healthcare challenge for medical communities globally.

Currently, many recent findings implicate soluble *β*-amyloid (A*β*s) but not the fibrillar aggregates contribute to the pathogenesis of AD. Soluble A*β* has demonstrated cytotoxic effects on neural cells, although the mechanism is not clear so far [2]. Proinflammatory cytokines are considered the possible candidates that mediated the cytotoxic effects of A*β* [3]. The consequent neuronal injury of neuroinflammation derived from A*β* includes axonal cytoskeleton breakdown and axonal swellings as well [4]. In the central nervous system (CNS), astrocyte is one of the major players which are responsible for producing cytokines locally [5]. The interaction between astrocyte and neuron in AD mouse model has been proved to be essential part of the AD pathogenesis and a promising target of new drug treatment [6]. Therefore, exploring the role of astrocyte in the A*β*-induced neuronal impairment is becoming very attractive during the years.

Irisin, also known as FNDC5, is a myokine which can induce “browning” of white adipose tissue and increase energy expenditure [7]. Irisin could ameliorate glucose metabolic derangements and might be promising novel therapeutic compound for diabetes mellitus (DM) in the future. Statistical analysis has revealed that about 70%–80% of AD patients may have DM or abnormal glucose metabolism [8]. Moreover, irisin is an active mediator for the beneficial effects of exercise on the CNS [9], which suggests that irisin could play an important therapeutic role in some brain disorders. Also, no effort is made to examine whether irisin can protect neuronal cells against A*β*-induced cytotoxicity.

In the present study, we want to investigate whether astrocyte is an active participator which mediates the neuroprotective effects of irisin. We found that irisin had no direct protective effects on neurons, but conditioned medium from irisin-pretreated astrocyte cultures could significantly attenuate the cytotoxicity of A*β*. Irisin was able to inhibit the release of IL-1*β* and IL-6 from astrocyte in cultures. The beneficial effects of irisin in astrocyte might be through the inhibition of NF*κ*B activation by reducing the phosphorylation of I*κ*B*α* and preventing the loss of total I*κ*B*α* in astrocyte exposed to A*β*.

## 2. Methods

### 2.1. Primary Cells and Drug Treatments

Cerebral hippocampal astrocytes were isolated from postneonatal pups of C57BL/6J mice as previously described [6, 10]. Cultures were incubated in a 95% humidified incubator at 37°C with 5% CO_2_. The astrocyte cultures were fed Dulbecco's modified Eagle's medium (DMEM, Life Technologies) with 10% fetal bovine serum (FBS, Life Technologies) every 3 days till they were 4 weeks old [6]. Hippocampal neurons were prepared from 16- to 18-day embryonic C57BL/6J mice as previously reported [6]. Hippocampal neurons were used for experiments on days 12–14 [6]. Irisin and A*β*_25–35_ were purchased from Sigma and was dissolved in culture media. The doses of these drugs for treatment were supported from previous literatures [11, 12].

### 2.2. Preparation of Astrocyte-Conditioned Medium (ACM)

Astrocyte cultures were incubated with irisin for 12 hours and then replaced with fresh medium. The ACM was collected 24 hours later. Control ACM (without irisin treatment) was collected with the same procedure. For the ACM treatment in neurons, 1 ml ACM and 1 ml fresh medium (1 : 1) were added to the neuron cultures as suggested by a previous report [13].

### 2.3. Cell Viability Assay

MTT assay was used to measure cell viability according to a previous report [7]. For cell viability study, neurons were plated in 96-well plates (2 × 10^5^ cells per well). MTT solution was added into the culture media with a final concentration of 10% in each well. After incubation for 4 h (37°C), 100 *μ*l of dimethylsulfoxide (DMSO) was added to each well for another 15 min. Then, the absorbance values were determined with a microplate reader (Thermo Fisher Scientific, Waltham, MA) at 570 nm.

### 2.4. Immunofluorescence Staining

Culture dishes were fixed with 4% paraformaldehyde for 30 min and then were treated with 0.2% Triton X-100 for another 30 min. After blocking with 3% bovine serum albumin for 2 h, primary antibodies to glial fibrillary acidic protein (GFAP) (Millipore, Burlington, MA) and microtubule-associated protein 2 (MAP2) (Millipore, Burlington, MA) were used overnight and followed by the detection of secondary antibodies (Invitrogen-Molecular Probes) for 2 hours. 1 *μ*g/ml Hoechst 33342 (Invitrogen-Molecular Probes) was used for nuclear staining.

### 2.5. Western Blot

Protein was extracted from cultured cells with cold lysis buffer containing phosphatase and protease inhibitor cocktail (Sigma). Total proteins were separated by electrophoresis with the 12% SDS-PAGE and then transferred onto nitrocellulose membranes (Thermo Fisher Scientific, Waltham, MA). The target proteins were measured using the primary antibodies of COX-2 (Abcam, Cambridge, United Kingdom), phosphorylated AKT (p-AKT, Cell Signaling, Danvers, MA), total AKT (t-AKT, Cell Signaling, Danvers, MA), NF*κ*B-p65 (Abcam, Cambridge, United Kingdom), phosphorylated I*κ*B*α* (p-I*κ*B*α*, New England Biolabs, Beverly, MA), total I*κ*B*α* (t-I*κ*Bα, New England Biolabs, Beverly, MA), and followed by detection with the secondary antibody and ECL kit (PerkinElmer, Waltham, MA). *β*-Actin (Santa Cruz, Dallas, TX) antibodies were used here as an internal control. Quantitative results are expressed as a ratio of target proteins to their internal control and phosphorylated proteins to their total proteins, accordingly.

### 2.6. Enzyme-Linked Immunosorbent Assay (ELISA)

The content of IL-1*β* and IL-6 in astrocyte medium was measured by using ELISA kits (BosterBio, Pleasanton, CA), according to the manufacturer's manual. For the ELISA test, astrocytes were plated in 96-well plate (Sigma, Saint Louis, MO) instead of petri dishes. Each treatment contained at least 6 wells, and the total medium from all these wells was mixed together. 200 *μ*l medium was loaded for each well in ELISA plate, and each sample was assayed in triplicate.

### 2.7. Statistical Analysis

GraphPad PRISM 5.0 software (GraphPad Software, La Jolla, CA) was used in the present study for statistical analysis. Comparison between groups was performed using analysis of one-way ANOVA followed by Newman–Keuls post hoc test or Student's *t*-test. Data are presented as mean ± S.E.M. Differences were considered statistically significant at *p* < 0.05.

## 3. Results

### 3.1. Irisin Protected Neuron in Cultures against A*β*-Induced Cell Viability Loss by Modulating Astrocytes

**Figure 1:**
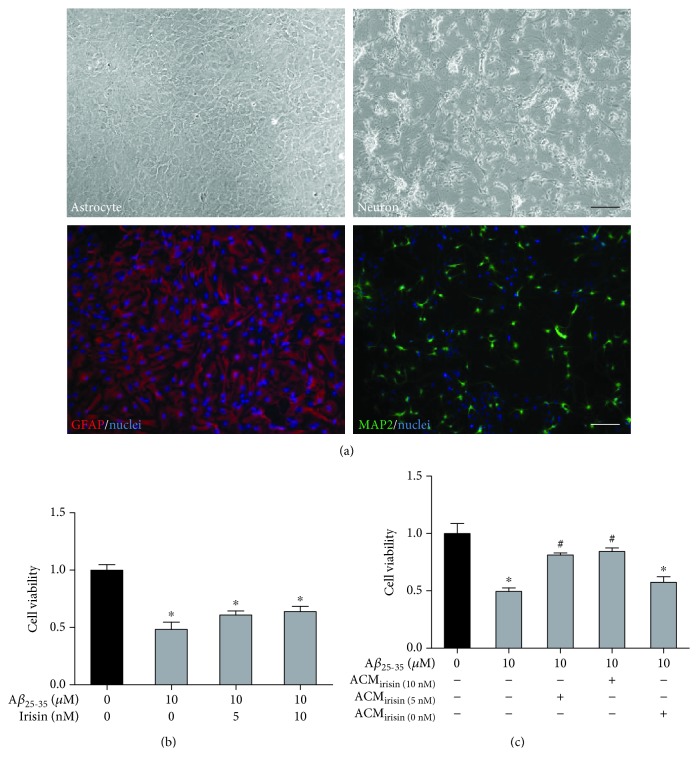
A*β*_25–35_ caused significant loss of cell viability in primary cultures of hippocampal neurons, which is not reversed by irisin cotreatment. (a) Representative phase contrast photographs of cultured hippocampal astrocytes (2 weeks old) and neurons (7 days). Representative immunofluorescent photographs of astrocyte markers GFAP and MAP2. Hoechst 33258 used as nuclei staining. Scale bar represents 100 *μ*M. (b) Irisin had no direct protective effects on the cell viability in neuron exposed to A*β*_25–35_ (MTT). (c) ACM_irisin_ exerted protective effects on the cell viability in neuron exposed to A*β*_25–35_ while control ACM without pretreatment of irisin had no such protective effects (MTT). Data are expressed as means ± SEM, ^∗^*p* < 0.05 vs control, ^#^*p* < 0.05 vs A*β*_25–35_, *n* = 6.

First, we set up cultures of hippocampal astrocytes and neurons, as shown in Figure 1(a). The purity of these cultured cells was identified with specific markers of GFAP and MAP2 for astrocyte and neuron, respectively. And then we asked whether irisin could exert neuroprotective effects on these culture models. We found that 10 *μ*M A*β* (25–35) treatment could cause significant loss of neuronal cell viability in 24 hours, but cotreatment of 5 or 10 nM irisin could not rescue the cell loss (Figure 1(b)). Next, we tested whether astrocyte could mediate the protective effects of irisin on neurons. Astrocyte cultures were incubated with irisin (0, 5, and 10 nM), and ACM was collected after 12 hours to incubate the hippocampal neurons for 30 minutes before A*β*_25–35_ was added. As shown in Figure 1(c), conditioned medium from irisin- (5 and 10 nM) pretreated astrocytes significantly improved the cell viability of neurons exposed to A*β*_25–35_ while control ACM showed no significant effect on the cell viability. The above results implied that irisin might exert its neuroprotective effect by modulating astrocytes.

### 3.2. Irisin Reduced the Released IL-1*β* and IL-6 Level in Cultured Astrocyte Exposed to A*β*_25–35_

**Figure 2:**
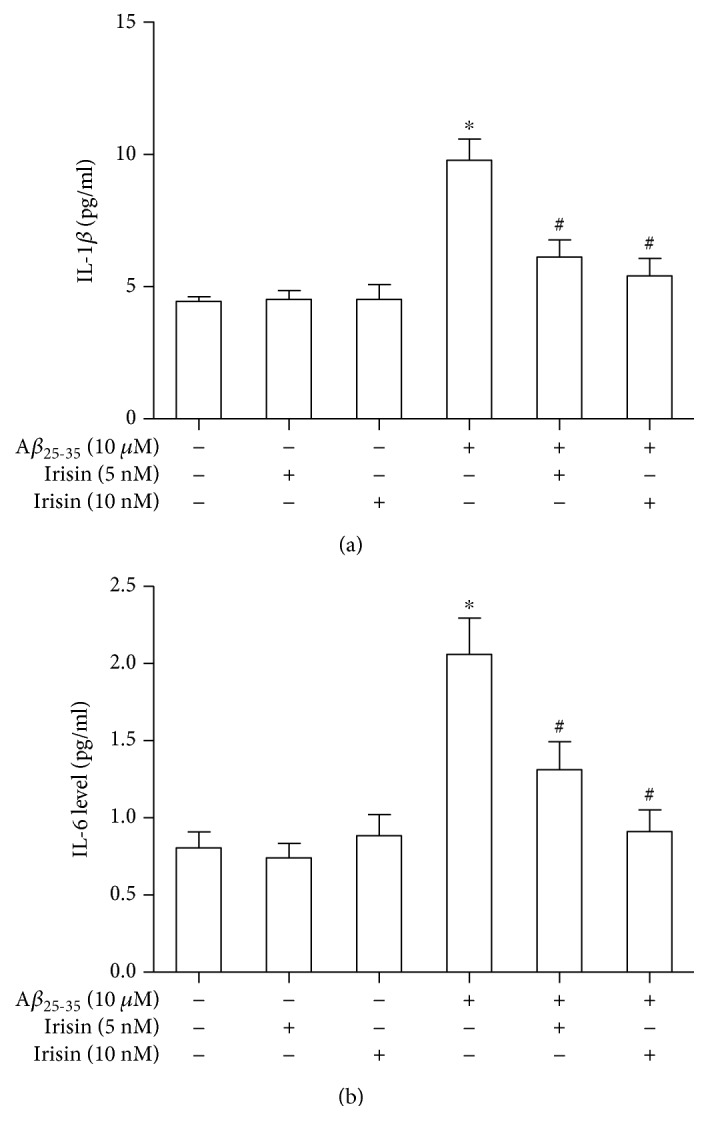
Irisin decreased the release of IL-1*β* and IL-6 from cultured astrocytes exposed to A*β*_25–35_. (a) Irisin could significantly prevent the A*β*_25–35_-induced IL-1*β* upregulation in ACM. (b) Irisin could significantly prevent the A*β*_25–35_-induced IL-6 upregulation in ACM. Data are expressed as means ± SEM, ^∗^*p* < 0.05 vs control, ^#^*p* < 0.05 vs A*β*_25–35_, *n* = 5.

Next, we hypothesized that irisin might inhibit the release of soluble factors which mediated the detrimental effects on neurons in cultures. Proinflammatory cytokines are a group of factors which are closely involved in the amyloid-induced cell injury in AD [14]. In the present study, we investigated two important cytokines, IL-1*β* and IL-6. ACM was collected after 12-hour irisin treatment, and ELISA experiments were carried out to measure the level of these two cytokines in the medium. As shown in Figure 2(a), A*β*_25–35_ treatment resulted in a significant increase of released IL-1*β* level in astrocyte cultures, which could be effectively decreased with cotreatment of irisin. And then we investigated the effects of irisin on the release of IL-6 in cultured astrocyte. We found that irisin could also obviously prevent the boost of released IL-6 level in astrocyte cultures exposed to A*β*_25–35_. These results suggested that irisin might attenuate the Aβ_25–35_-induced neuronal cell loss by inhibiting the release of IL-1*β* and IL-6 from astrocyte.

### 3.3. Irisin Reduced the Inducible Expression of Cyclooxygenase-2 (COX-2) and Phosphorylation of AKT in Cultured Astrocyte Exposed to A*β*_25–35_

Induction of COX-2, a proinflammatory factor, is involved in inflammatory response [15]. We postulated that COX was a possible candidate protein which was regulated by irisin to attenuate the inflammation in astrocyte exposed to A*β*_25–35_.

**Figure 3:**
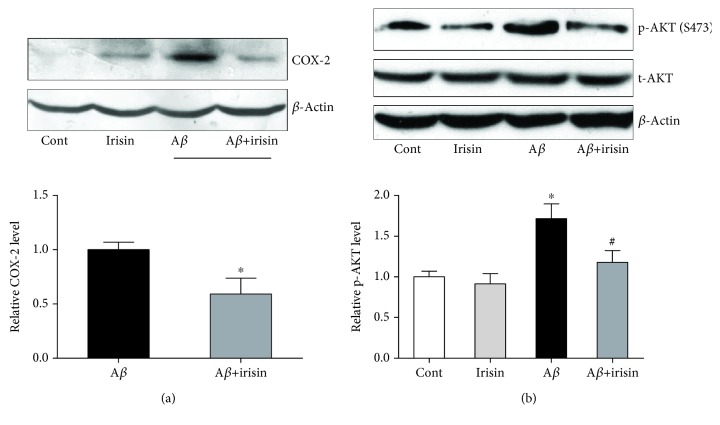
Irisin decreased the increased expression of COX-2 and p-AKT in cultured astrocytes exposed to A*β*_25–35_. (a) Representative picture and statistical result showing irisin decreased the COX-2 level in astrocytes exposed to A*β*_25–35_. (b) Representative picture and statistical result showing irisin decreased the p-AKT level in astrocyte exposed to A*β*_25–35_. Data are expressed as means ± SEM, ^∗^*p* < 0.05 vs control, ^#^*p* < 0.05 vs A*β*_25–35_, *n* = 5.

As shown in Figure 3(a), COX-2 demonstrated an inducible expression in astrocyte treated with A*β*_25–35_, but the upregulation was prevented by cotreatment with irisin. Another key protein in inflammation, AKT, was investigated with Western blot to measure the phosphorylation of this protein in the following Western blot study. We found that p-AKT (S473) was significantly upregulated in cultured astrocytes with A*β*_25–35_ treatment compared to total AKT, which was able to be partially reversed by the cotreatment of irisin (Figure 3(b)).

### 3.4. Irisin Decreased Phosphorylation and Degradation of I*κ*B*α* Leading to NF*κ*B Lower Activation in Astrocyte

**Figure 4:**
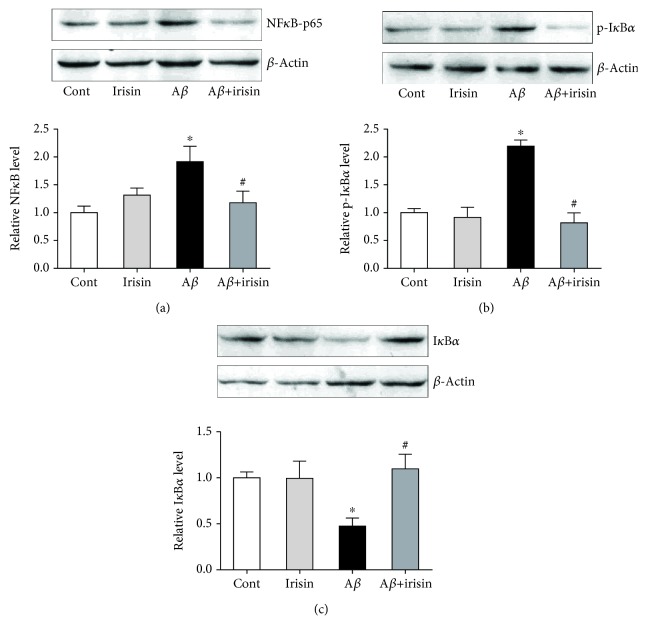
Irisin decreased NF*κ*B activity by preventing the phosphorylation and loss of I*κ*B*α* in cultured astrocytes exposed to A*β*_25–35_. (a) Representative picture and statistical result showing irisin decreased the NF*κ*B level in astrocytes exposed to A*β*_25–35_. (b) Representative picture and statistical result showing irisin decreased the p-I*κ*B*α* level in astrocytes exposed to A*β*_25–35_. (c) Representative picture and statistical result showing irisin increased the total I*κ*B*α* level in astrocytes exposed to A*β*_25–35_. Data are expressed as means ± SEM, ^∗^*p* < 0.05 vs control, ^#^*p* < 0.05 vs A*β*_25–35_, *n* = 5.

We further explored the possible mechanism by which irisin regulated the COX-2 and proinflammatory factors. We assessed the protein expression level of NF*κ*B (p65) in astrocyte cultures, and our results showed that NF*κ*B protein level was increased after A*β*_25–35_ treatment but irisin could effectively decrease the upregulated expression (Figure 4(a)). Moreover, the phosphorylation of I*κ*B*α* (p-I*κ*B*α*), a NF*κ*B inhibitor protein, was also significantly upregulated in astrocyte after A*β*_25–35_ treatment although it could be prevented by irisin cotreatment (Figure 4(b)). Accordingly, total I*κ*B*α* protein expression level in cultured astrocytes was reduced (Figure 4(c)). Collectively, these Western blot results implied that irisin not only directly decreased the NF*κ*B expression level but also reduced the phosphorylation and the loss of total I*κ*B*α*, which might prevent the release of NF*κ*B from I*κ*B*α* in astrocyte and finally inhibit the level of cytokines released from astrocyte.

## 4. Discussion

AD is one of the most devastating diseases among dementia. Recent studies have supported the idea that soluble A*β* might be the culprit of neuronal cell injury and synaptic impairment in AD [16]. Reducing soluble A*β* rather than insoluble A*β* improved the memory and cognitive performance in transgenic mice of AD with plaques [17]. Therefore, preventing the soluble A*β* cytotoxicity to neurons may be another promising strategy to treat AD.

In the present study, we carried out experiments on cultured astrocytes and neurons to investigate whether an exercise hormone could prevent the cytotoxicity of soluble A*β*. Astrocytes have been found to be a potential target for the development of neuroprotective drugs for many years [6]. Consistent with previous studies, we found that soluble A*β* treatment could cause the loss of neuronal cell viability in cultures. To prevent cell injury, we introduced irisin into the cell culture model to see whether this myokine could protect neurons from A*β* injury. Our results showed that irisin exerted its protective effects on neurons via an astrocyte-dependent way. Irisin had no direct protective effects on cultured neurons but was able to significantly reduce the release of cytokines from astrocytes. Moreover, this resulted in notable neuroprotection of the astrocyte-conditioned medium on hippocampal neurons in cultures. This suggests that astrocytes are essential parts of the protective effects of irisin on neurons in AD.

As mentioned above, cytokines have been found to play an important role in A*β*-induced neuropathology of AD. As astrocytes are major producers of these cytokines within the CNS, we asked whether irisin regulated the release of cytokines from astrocytes during its protective effects on neurons. IL-1*β* and IL-6 were investigated in the present study, and we found that irisin could significantly prevent the A*β*-induced increase of these two cytokines of astrocyte culture medium. The reduction of IL-1*β* and IL-6 might be responsible for the consequent beneficial effects of conditioned medium on neurons. Next, we tried to decipher the possible underlying mechanisms which are responsible for the above changes. COX-2 can be induced by proinflammatory stimuli in the CNS [18]. Therefore, we explored whether COX-2 and another important player in inflammation, AKT, were regulated during the treatment in cultures. As expected, our results demonstrated that irisin inhibited the expression level of these two proteins in astrocytes exposed to A*β*. Since the promoter area of COX-2 gene includes sequences that can be responsive to regulation by NF*κ*B [19], we also tested whether NF*κ*B was involved in the regulation. We found that this transcriptional factor was significantly upregulated by A*β* treatment but irisin cotreatment could effectively reduce the increased expression level in astrocytes. Since I*κ*B*α* could inhibit NF*κ*B activity through sequestration in the cytoplasm [15], the expression level of I*κ*B*α* protein was explored in the present study, and we found that A*β* caused enhanced phosphorylation of I*κ*B*α*, which led to the loss of total I*κ*B*α*. The reduction of total protein level of I*κ*B*α* enhanced the release of NF*κ*B and boosted the level of IL-1*β* and IL-6 from astrocytes. However, irisin cotreatment could reduce the NF*κ*B level in astrocytes after A*β* stimulation. Moreover, irisin could increase the I*κ*B*α* level and then prevent the release of NF*κ*B. According to our knowledge so far, our study is the first to show that irisin can exert anti-inflammatory effects on astrocytes in the CNS. Astrocytes have been proved to be the most abundant cell population in the CNS and greatly outnumber neurons [20]. Astrocytes are becoming very important player for the treatment in many diseases and closely involved in memory and cognition deficits in DM [21–23]. Therefore, irisin may be a promising candidate for the treatment of DM, especially in elderly patients. Our finding may not only open a new route for the treatment of AD but may also provide some evidences to develop novel compounds which target astrocyte in the CNS.

## 5. Conclusion

Our results in this study implied that the neuroprotection of irisin was mediated by blocking the release of Il-1*β* and IL-6 from astrocyte instead of its direct action on neurons. Our results also suggested that NF*κ*B signaling pathway played an important role in the regulation of irisin on astrocytes exposed to A*β*. While disclosing an important role of astrocytes in A*β* pathogenesis of AD, our studies also provided new evidence for the idea that irisin might be a promising compound for the treatment AD and DM.

## Data Availability

The data used to support the findings of this study are available from the corresponding author upon request.
